# Harnessing the Power of Machine Learning Guided Discovery of NLRP3 Inhibitors Towards the Effective Treatment of Rheumatoid Arthritis

**DOI:** 10.3390/cells14010027

**Published:** 2024-12-30

**Authors:** Sidra Ilyas, Abdul Manan, Chanyoon Park, Hee-Geun Jo, Donghun Lee

**Affiliations:** 1 Department of Herbal Pharmacology, College of Korean Medicine, Gachon University, 1342 Seongnamdae-ro, Sujeong-gu, Seongnam-si 13120, Republic of Korea; sidrailyas6@gachon.ac.kr (S.I.);; 2 Department of Molecular Science and Technology, Ajou University, Suwon 16499, Republic of Korea; mananriaz012@gmail.com; 3 Naturalis Inc. 6, Daewangpangyo-ro, Bundang-gu, Seongnam-si 13549, Republic of Korea

**Keywords:** NLRP3, machine learning, QSAR, docking, MD simulation, rheumatoid arthritis

## Abstract

The NLRP3 inflammasome, plays a critical role in the pathogenesis of rheumatoid arthritis (RA) by activating inflammatory cytokines such as IL1β and IL18. Targeting NLRP3 has emerged as a promising therapeutic strategy for RA. In this study, a multidisciplinary approach combining machine learning, quantitative structure–activity relationship (QSAR) modeling, structure–activity landscape index (SALI), docking, molecular dynamics (MD), and molecular mechanics Poisson–Boltzmann surface area MM/PBSA assays was employed to identify novel NLRP3 inhibitors. The ChEMBL database was used to retrieve compounds with known IC_50_ values to train machine learning (ML) models using the Lazy Predict package. After data pre-processing, 401 non-redundant structures were selected for exploratory data analysis (EDA). PubChem and MACCS fingerprints were used to predict the inhibitory activities of the compounds. SALI was used to identify structurally similar compounds with significantly different biological activities. The compounds were docked using MOE to assess their binding affinities and interactions with key residues in NLRP3. The models were evaluated, and a comparative analysis revealed that the ensemble Random Forest (RF) model (PubChem fingerprints) with RMSE (0.731), R^2^ (0.622), and MAPE (8.988) and bootstrap aggregating model (MACCS fingerprints) with RMSE (0.687), R^2^ (0.666), and MAPE (9.216) on the testing set performed well, in accordance with the Organization for Economic Cooperation and Development (OECD) guidelines. Out of all docked compounds, the two most promising compounds (ChEMBL5289544 and ChEMBL5219789) with binding scores of −7.5 and −8.2 kcal/mol were further investigated by MD to evaluate their stability and dynamic behavior within the binding site. MD simulations (200 ns) revealed strong structural stability, flexibility, and interactions in the selected complexes. MM/PBSA binding free energy calculations revealed that van der Waals and electrostatic forces were the key drivers of the binding of the protein with ligands. The outcomes obtained can be used to design more potent and selective NLRP3 inhibitors as therapeutic agents for the treatment of inflammatory diseases such as RA. However, concerns related to the lack of large datasets, experimental validation, and high computational costs remain.

## 1. Introduction

**Figure 1:**
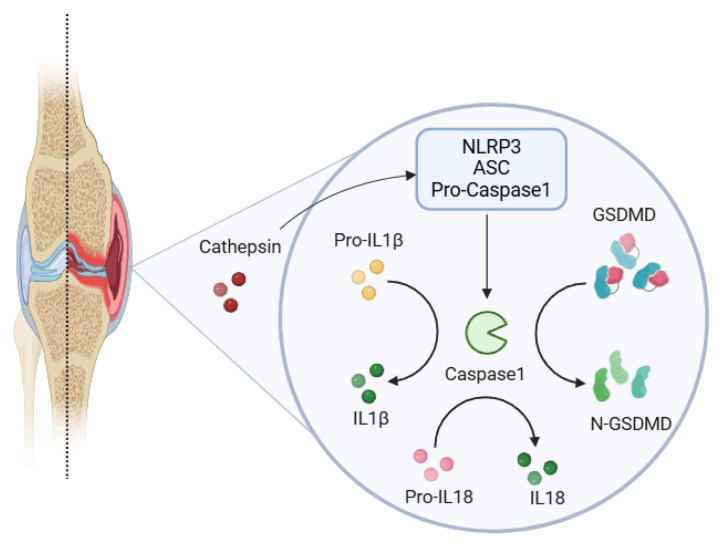
Cathepsin release from bone activates NLRP3, which triggers caspase1 to cause cleavage of gasdermin D (GSDMD), and release of inflammatory cytokines (IL1β and IL18) leading to joint inflammation in RA.

Rheumatoid arthritis (RA) is a chronic autoimmune disease characterized by persistent joint inflammation and destruction. The pathogenesis of RA involves a complex interplay between immune dysregulation and inflammatory processes. Key factors contributing to RA include an imbalance of T helper 17 (Th17) and regulatory T (Treg) cells, as well as activation of the NLRP3 inflammasome involved in NF-kB signaling pathways [1]. The NLRP3 multiprotein complex, comprising the NACHT, LRR, and PYD domains, plays a critical role in the innate immune response [2]. NLRP3, when bound to ADP, is suppressed under resting conditions; nonetheless, it becomes activated upon several stimuli, such as pathogens, reactive oxygen species (ROS), potassium efflux, and cathepsin release by the bone [3,4]. Upon activation, it forms an inflammasome polymeric complex composed of NLRP3, caspase1 (CASP1), and PYCARD/ASC, which subsequently activates caspase1-dependent cell death, called pyroptosis [5]. Pyroptosis causes a quick rupture of the plasma membrane and the release of proinflammatory cytokines, especially IL1β and IL18, leading to the pathophysiology of RA [6,7,8]. The expression of NLRP3 inflammasome complex triggers gasdermin D (GSDMD) cleavage to produce a caspase-matured N-terminal fragment, forming pore-like structures for the release of lactate dehydrogenase (LDH), IL1β, and IL18 as shown in Figure 1 [9]. IL1β induces bone resorption by upregulating RANKL expression and stimulating osteoclast differentiation [10]. It also contributes to cartilage degradation by inducing the production of matrix metalloproteinases (MMPs) through the activation of NF-kB and MAPK signaling pathways. NF-kB, a transcription factor, plays a pivotal role in regulating the expression of inflammatory genes involved in RA [11]. IL18 further amplifies inflammation by activating T cells and promoting the production of proinflammatory cytokines. Animal models of collagen-induced arthritis and antigen-induced arthritis have demonstrated the involvement of NLRP3 in the development and increased susceptibility of joint inflammation in RA [12,13].

Consequently, targeting the NLRP3 inflammasome and its components involved in key pathways represents a promising therapeutic strategy for the treatment of RA [14]. Inhibiting NLRP3 activation and reducing the upregulation of IL1β and IL18 can alleviate inflammation and joint damage. Several NLRP3 inhibitors have been developed to target the NLRP3 inflammasome, including MCC950, VX-765, and parthenolide [15,16]. Although most of these compounds have shown promise in preclinical studies, they often lack selectivity and can exert off-target effects [15].

Quantitative structure–activity relationship (QSAR) modeling is a powerful tool for determining the physicochemical properties of compounds associated with their bioactivity. Classical QSAR statistical methods, such as linear regression or partial least squares (PLS), are often used to analyze data. Currently, machine learning (ML) and deep learning (DL) are applied to process and enhance traditional QSAR models. While traditional QSAR methods focus more on linear or semi-linear relationships, ensemble-based ML approaches, such as Gradient Boosting (GB), bootstrap aggregating, LightGBM, Histogram Gradient Boosting (HGB), and Random Forest (RF), can capture more complex, nonlinear patterns in data. These ML techniques handle large, high-dimensional datasets and can improve prediction accuracy by learning from more intricate relationships in the data. QSAR models built on cutting-edge ML algorithms for assessing the chemical spaces of molecular descriptors using various validation techniques are complex. According to the similarity-property principle (SPP), biological activity is determined by compound structural similarity, and similar structural compounds have similar biological properties and activities [17]. QSAR modeling has five guidelines established by the Organization for Economic Cooperation and Development (OECD): (1) defined endpoint, (2) clear methodology, (3) defined applicability domain (AD), (4) modeling validation, and (5) mechanistic interpretation.

This study aimed to predict the bioactivity of NLRP3 inhibitors using data retrieved from the ChEMBL database. ChEMBL is a publicly accessible database of small compounds with known drug-like properties that provides detailed information on their chemical structures, biological activities, and pharmacological properties. The chemical structures of the NLRP3 inhibitors were analyzed using extremely powerful ML tools. Five machine learning regression algorithms were established to build QSAR models to predict the inhibitory bioactivity of the compounds against NLRP3. The predictability and robustness of the best model were assessed. The structure–activity landscape index (SALI) was used to identify structurally similar compounds with significantly different biological activities. Docking, molecular dynamics (MD) simulations, and molecular mechanics/Poisson–Boltzmann surface area (MM/PBSA) analyses were performed. By combining classical and ML modeling techniques, the optimization process for further NLRP3 inhibitors can be guided, potentially leading to treatments for a variety of inflammatory disorders, including RA.

## 2. Experimental Procedures

**Figure 2:**
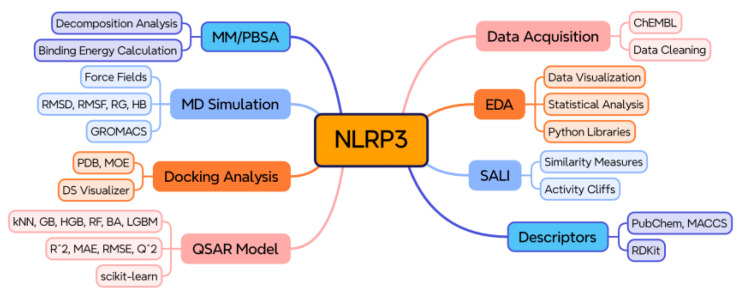
Research workflow of the study conducted.

The research workflow consisted of data collection, data pre-processing, exploratory data analysis (EDA), QSAR modeling, SALI, docking, MD simulation, and MM/PBSA as displayed in Figure 2.

### 2.1. Data Acquisition and Preparation

A dataset of compounds with known biological activity (IC_50_ values) was obtained from the ChEMBL database (ChEMBL ID: 1741208). The dataset was pre-processed to remove duplicate compounds, and missing and conflicting IC_50_ activity values were eliminated to ensure data quality. Since the bioactivity dataset of NLRP3 inhibitors was denoted by IC_50_, pIC_50_ was generated by taking the negative logarithm to the base of 10, the bioactivities of compounds were partitioned into two categories: active and inactive.

### 2.2. Exploratory Data Analysis

Lipinski’s rule of five is used for identifying drug-like qualities of molecules and based on physicochemical properties, six molecular descriptors including molecular weight (MW), octanol–water partition coefficient (LogP), number of hydrogen bond acceptors/donors (nHA/HD), number of rotatable bonds (nRot), and topological polar surface area (TPSA), which were calculated using the RDKit cheminformatics [18,19]. Analysis of the chemical space (theoretical space where a molecule exists) was performed to comprehend the general characteristics of active and inactive groups.

### 2.3. Statistical Analysis

Statistical analyses were performed to compare the physicochemical properties of the different groups of compounds. Univariate statistical analysis, the nonparametric Mann–Whitney U test (to evaluate the dependent variable’s distribution), and Student’s *t*-test were applied to determine significant differences between the active/inactive groups. PCA, an unsupervised machine learning technique, was used to reduce the dataset dimensionality. The PCA bounding box approach was used for AD visualization of the model [20]. The data matrix for PCA was structured such that each row represented a compound with biological activity (pIC_50_), and each column corresponded to a specific molecular descriptor. This matrix was used to reduce dimensionality and identify key patterns and features in the data.

### 2.4. Similarity Activity Landscape Analysis

PubChem and MACCS were the molecular fingerprints used to generate the SAS maps and activity cliffs. Activity Landscape Plotter V.1 (UNAM, Mexico) “URL http://132.248.103.152:3838/ActLSmaps/ (accessed on 7 July 2024)” a webserver to generate SAS maps, was used, and the thresholds of structure and activity similarity were set to 0.9 and 2, respectively [21]. SAL analysis was performed to identify pairs of molecules with high structural similarity but significant differences in activity. The structural similarity between the molecules was calculated using a suitable similarity metric (Tanimoto similarity). The difference in activity was calculated as the absolute difference in pIC_50_ values. The distribution patterns of Murcko and cyclic skeleton scaffolds were identified, compared using pIC_50_ levels, and further analyzed [22]. The frequencies of scaffolds and skeletons were calculated and ranked using DataWarrior (version 5.5.0) [23].

### 2.5. Molecular Descriptor Generation

Molecular fingerprints are advanced representations of small molecules that characterize their structure, interconnectivity, similarities, and physical and chemical properties through bit-string comparisons. The free open-source PaDEL software (version 2.21) tool offers PubChem fingerprints, which use 881 binary (1/0) bits as chemical characteristics to indicate whether a particular group is present in a compound, and MACCS fingerprints, which comprise 166 bits associated with SMARTS patterns [24].

### 2.6. Feature Selection

In order to avoid overfitting/bias and to increase the QSAR model’s accuracy, which could negatively impact the outcomes, feature selection was carried out. To ascertain which features were most relevant for predicting bioactivity and to reduce feature complexity, a correlation-based filter method and Random Forest regressor model were employed. Features with collinearity (correlation > 0.90) and low variance (variance < 0.1) were removed [25]. This level of dimensionality reduction aims to improve interpretability and reduce computational costs while maintaining the most informative features of the model.

### 2.7. QSAR Model Construction and Validation

A dataset of compounds with known biological activity was used to compute molecular descriptors representing structural features, and machine learning algorithms were applied to correlate the descriptors with biological activity. Five QSAR regression models were independently utilized for model construction, which has shown great potential for predicting the inhibitory biological activity of a given compound, thereby reducing the cost and time required for drug development. Several ML models for predicting pIC_50_ values of compounds have been developed. kNN, GB, bootstrap aggregation, LightGBM, HGB, and RF were used. RF is a standard ensemble bootstrapping technique consisting of many decision trees that randomly select a subset of features at each node [26]. Through averaging, it analyzes the forecast from several trees and generates the final result based on the majority of anticipated outcomes. Another ensemble learning method is GB, which combines multiple weak decision trees and enhances the performance by adding more trees to fix mistakes committed by earlier trees by calculating residuals. High-performance models are frequently produced using this iterative procedure, particularly for complicated datasets.

The leafwise tree growth approach used by the LightGBM was more effective than the conventional depthwise approach. To further enhance the model efficiency and performance, two methods are used: gradient-based sampling and exclusive feature building [27]. Another Gradient Boosting method that approximates the feature distribution using histograms is Histogram Gradient Boosting. This method works well with large datasets because it can significantly lower computational costs and memory usage [28]. One of the earliest and most basic kNN algorithms was developed in the early 1970s and uses a linear decomposition methodology to operate in the coordinate plane, which clusters objects based on their relatedness to one another [29,30]. It calculates the cosine, Hamming, and Euclidean distances between data points to obtain neighboring data. Although it is easy to understand and implement, kNN can be computationally expensive for large datasets. These models were trained using a training set and evaluated using a test set. The performance of each model was assessed, and the algorithm that produced the best results was selected. The performance of each model was assessed using metrics such as RMSE, R^2^, Q^2^, and MAPE.

### 2.8. Model Validation

A 4:1 ratio was used to divide the active and inactive compounds into training and test sets, respectively. The predictive ability of the QSAR model was verified using the Y-scrambling test, external validation, and 10-fold cross-validation [31]. The dataset was randomly divided into ten folds using the ten-fold cross-validation method; nine of these folds were utilized for model training, and one was used for testing. This procedure was carried out ten times, with each time setting aside a distinct fold for testing. The performance of the prediction model was assessed using the average accuracy. Model validation is essential to ensure that a fitted model can correctly forecast unknown data. To evaluate the effectiveness of the QSAR models, two statistical metrics were employed: the RMSE and Pearson correlation coefficient (r). For describing the strength of the relationship between two variables of interest, the Pearson correlation coefficient, which has a range of −1 (negative correlation) to +1 (positive correlation), was used. The relative error of the prediction models was analyzed by RMSE, whereas the MAPE was calculated to further analyze the model’s performance and prediction accuracy on the datasets; a lower MAPE indicates better model performance.

The MAPE calculations are as follows:

$$MAPE=1/N\sum_{k=1}^{N}\frac{\left|Xk-\hat{X}k\right|}{Xk}\times 100\%$$

where Xk is the actual value, $\hat{X}$k is the predicted value of the model, |Xk−$\hat{X}$k| is the absolute error, and N is the number of incidence data points.

### 2.9. Molecular Docking Analysis

Protein–ligand docking was performed using the MOE platform (version 2020.09) “URL https://www.chemcomp.com/en/index.htm (accessed on 23 July 2024)” to investigate the binding modes of ligands to NLRP3 [32]. The NLRP3 structure in complex (PDB ID: 7alv) was obtained from the Protein Data Bank (PDB) database (USA), “URL https://www.rcsb.org/ (accessed on 7 August 2024)”, and protein and ligands were prepared using MOE. The NACHT site of NLRP3 was selected for docking, and the grid box was set to the X, Y, and Z axes at 17.60, 34.32, and 125.09, respectively. The previously attached ligand was removed from the NLRP3 structure before docking to render the active sites available for new ligands. To avoid obscuring the contact site during docking, water molecules, salts, and other undesirable compounds were eliminated. Subsequently, the MOE structure preparation module was used to correct the errors, add missing atoms, and correct the NLRP3 structure. Standard protocols and settings were used for docking. Key interactions that contribute to ligand binding, including hydrophobic interactions, π-π interactions, and hydrogen bonds, were identified by analyzing the resultant protein–ligand complexes. The Discovery Studio program, which offers a two-dimensional representation of protein–ligand interactions, was used to visualize the docking results.

### 2.10. MD Simulation

MD simulations were performed to investigate the dynamic behavior of protein–ligand complexes. The docked complexes were modeled using the CHARMM36 all-atoms force field and then embedded using the TIP3p water model in a cubic water box with a 12 Å buffer distance [33]. The CGenFF server was used to build the ligand topology, which was then adapted for compatibility with Gromacs simulation software “URL https://cgenff.com/ (accessed on 14 August 2024)”. The simulation system was neutralized by adding appropriate numbers of sodium (Na^+^) and chloride (Cl^−^) ions. Energy minimization was conducted using the steepest descent algorithm until the system reached a gradient tolerance of 0.001 kJ/mol. To achieve equilibration, the systems were subjected to temperature and pressure control using the Nose–Hoover and Parrinello–Rahman methods, respectively [34]. Short-range electrostatic and van der Waals interactions were calculated within a cut-off radius of 1.2 nm, whereas long-range electrostatic interactions were treated using the particle mesh Ewald method [35]. For every complex, 200 ns MD simulations were performed using the Gromacs 2024.1 program “URL https://www.gromacs.org/ (accessed on 14 August 2024)”. The resulting trajectories were examined using Gromacs built-in tools, and structural visualizations were produced [36].

### 2.11. MM/PBSA Binding Free Energy of the Protein–Ligand Complexes

To further elucidate the energetic contribution to ligand binding, MM/PBSA calculations were performed using a representative set of MD snapshots [37,38]. The binding free energy (∆Gbind) was decomposed into individual energy terms: van der Waals (∆EvdW), electrostatic (∆Eelec), polar solvation (∆Gpol), and nonpolar solvation (∆Gnpolar). Additionally, per-residue decomposition analysis was conducted to identify the key residues involved in ligand recognition and binding. This analysis revealed that the residues contributed significantly to the overall binding affinity, highlighting the importance of chemical interactions in stabilizing the protein–ligand complex.

The MM/PBSA method calculates the binding free energy (∆Gbind) as follows:

$${∆G}_{bind}={∆E}_{vdW}+{∆E}_{elec}+{∆G}_{solv}-T∆S$$

where ∆EvdW is van der Waals interaction energy; ∆Eelec is electrostatic interaction energy; ∆Gsolv represents solvation free energy, which can be further decomposed into polar (∆Gpol) and nonpolar (∆Genpolar) contributions; and T∆S shows entropic contribution, often neglected in MM/PBSA calculations. The per-residue decomposition analysis allows for the identification of key residues contributing to the binding affinity. This is achieved by calculating the energy contributions for each residue in the protein–ligand complex and comparing them to the corresponding contributions in the unbound protein and ligand. Residues with significant energy differences are likely to play a critical role in ligand binding. By combining MM/PBSA calculations with per-residue decomposition analysis, it is possible to gain insights into the molecular mechanisms underlying protein–ligand interactions and to identify potential targets for drug design.

## 3. Results

### 3.1. Exploratory Data Analysis

The dataset was initially retrieved from the ChEMBL database (version 31; target ID: 1741208). A total of 529 compounds against human NLRP3 protein were selected based on the IC_50_ (compound concentration required for 50% inhibition). IC_50_ was converted into pIC_50_ (negative logarithm of IC_50_) to compare the potency of different categories of inhibitors at the same molar level. After data was cleansed and pre-processed, the working dataset consisted of non-redundant unique smiles of 402 compounds, of which 179 were classified as active with pIC_50_ values greater than 5.5, and 226 were regarded as inactive with pIC_50_ values less than 5.5. The nonparametric Mann–Whitney U test revealed distribution patterns for MW, LogP, nHA, nHD, nRot, and TPSA, with *p*-values < 0.05. Four attributes (MW, nHA, nHD, and TPSA) confirmed statistically significant physiochemical differences between the active and inactive compounds, whereas no significant differences were observed in LogP and nRot.

**Figure 3:**
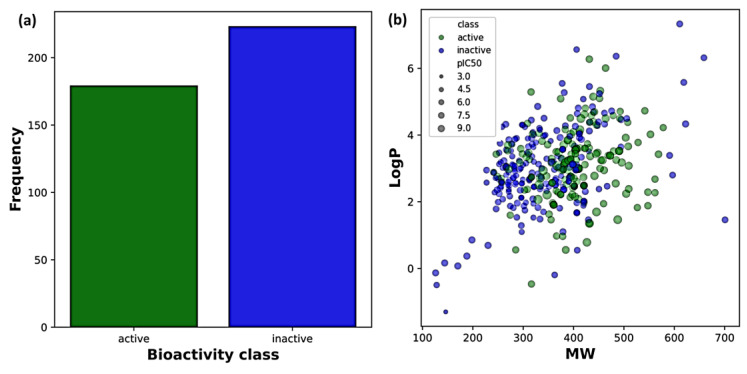
pIC_50_ and chemical space of molecules. (**a**) pIC_50_ of active and inactive compounds; (**b**) scatter plot of molecular weight (MW) vs. octanol–water partition coefficient (LogP) indicating the chemical space occupied by active (green) and inactive (blue) compounds.

**Figure 4:**
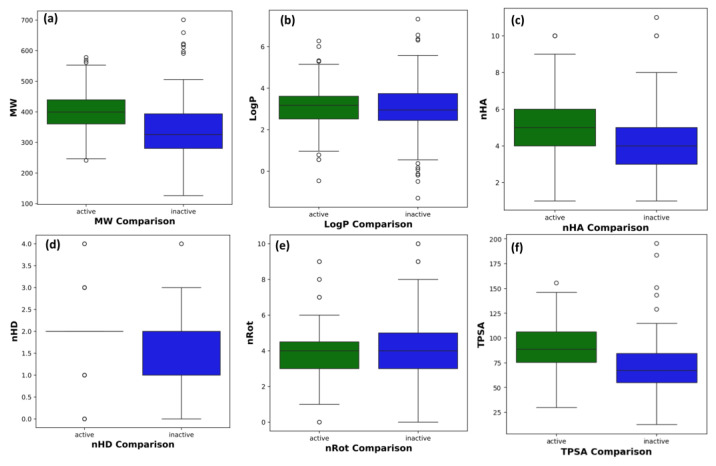
Box plots of physicochemical properties of drug-likeness between active and inactive compounds. (**a**–**f**) were showing the six distribution patterns of MW, LogP, nHA, nHD, nRot, and TPSA.

Statistical analysis revealed significant differences in distribution patterns between the two groups (Table 1). Both groups showed relatively low values of hydrogen bond donors/acceptors, with the inactive compounds exhibiting slightly higher average values. Notably, the active compounds had a significantly higher number of rotatable bonds and larger TPSA, suggesting greater flexibility and polarity. The chemical spaces of the active and inactive compounds and the scatter plots of MW and LogP are shown in Figure 3. Chemical space visualization using six physicochemical properties demonstrated that the active compounds displayed a broader range of MW and LogP values, indicating greater diversity and lipophilicity than inactive compounds (Figure 4).

### 3.2. Principal Component Analysis

Principal component analysis (PCA) was performed to reduce the dimensionality of the dataset and identify the most significant physicochemical properties that influence biological activity. The first three principal components accounted for 84% of the total variance in the dataset. PC1, with an eigenvalue of 0.44, was primarily influenced by MW and TPSA, suggesting that these properties contributed significantly to the overall variance. PC2, with an eigenvalue of 0.25, was strongly correlated with LogP and nHA, indicating that these properties play crucial roles in the second dimension of variation. PC3, with an eigenvalue of 0.15, was mainly influenced by nHD and nRot, suggesting its importance in determining the remaining variance (Table 2).

### 3.3. Solvent Accessible Surface Maps and SALI

**Figure 5:**
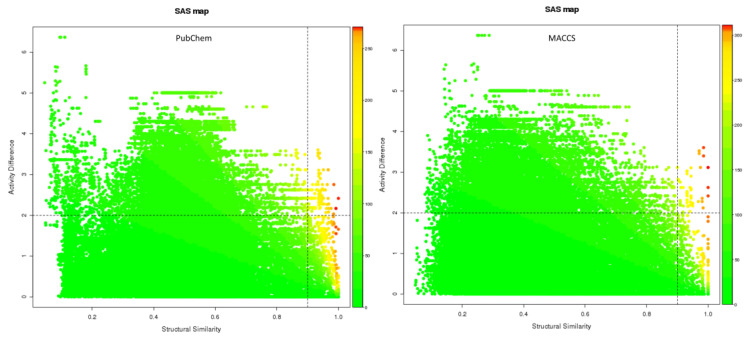
Comparison of solvent accessible surface (SAS) maps using PubChem and MACCS fingerprints with activity cliffs quadrant set as X > 0.9 and Y > 2. The gradual transformation of color from green to red indicates a steady increase in the SALI value.

In PubChem, the pair (ChEMBL5219210_ChEMBL5219789) with the highest SALI value had a similarity score of 1, indicating that the two compounds were structurally highly similar (Figure 5). However, they exhibited significant differences in activity, suggesting that a small structural modification can lead to a large change in biological activity, which can be used to design new compounds with improved properties. For MACCS, three pairs (ChEMBL5180041_ChEMBL5196600, ChEMBL5186980_ChEMBL5182226, and ChEMBL5219210_ChEMBL5219789) showed the highest SALI values, with a similarity score of 1 (Supplementary Table S1). In addition, the Murcko scaffolds with frequencies of 7, 10, 11, and 12 have a common tricyclic skeleton and other structural features, including aromatic rings, heterocyclic systems, and functional groups such as amines, amides, and carboxylic acids (Supplementary Figures S1 and S2). The scaffold with the highest frequency (41) showed varying levels of structural enrichment (Table 3).

### 3.4. QSAR Modeling and Validation of NLRP3 Inhibitors

The effectiveness of the machine learning models in predicting pIC_50_ values was assessed using the lazy regressor approach. A dataset containing features with pIC_50_ values from the training set was used to train the models. Training and testing sets were used to evaluate the performance of each model. The top five regression algorithms were built for each PubChem and MACCS fingerprint to predict the bioactivity classes of NLRP3 inhibitors and were implemented using sklearn (Supplementary Tables S2 and S3) [39]. A comprehensive analysis of various machine learning models was used to predict the pIC_50_. For the MACCS fingerprints, GB, bootstrap aggregation, LightGBM, HGB, and RF were trained and evaluated (Supplementary Table S3). Hyperparameter tuning was performed, and the random state was set to 42 for reproducible results (Supplementary Table S4). The Y-scrambling test showed experimental and predictive measurements of the QSAR model (Supplementary Figure S3). Metrics, including the root mean square error (RMSE), R^2^, Q^2^, and mean absolute error (MAPE), were used to evaluate the performance of each model. With the lowest RMSE and MAPE and highest R^2^ values for both the training and testing sets, bootstrap aggregation was considered the best-performing model (Table 4). In terms of PubChem fingerprints, GB, kNN, LightGBM, HGB, and RF were evaluated. RF, with the lowest MAPE (8.9%) on the test data, was considered the best-performing model, whereas LightGBM and HGB showed similar metric performances (Table 4).

**Figure 6:**
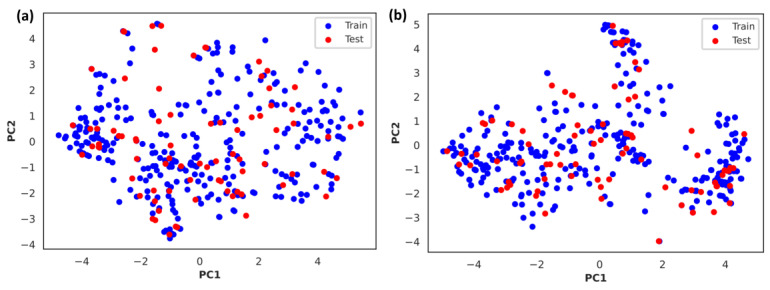
Applicability domain (AD) visualization of (**a**) PubChem fingerprints and (**b**) MACCS fingerprints by PCA. The distribution of the training (blue circles) and test (red circles) datasets in the chemical space is represented. The two datasets are dispersed in the chemical space over the same area, and the test set falls within the AD of the model, indicating the model’s prediction efficiency.

The set of circumstances under which a model is created to predict unknown compounds using the training set is known as the AD in machine learning, which is used to assess the predictive reliability of the model [17]. PCA of the test dataset revealed that it fell within the range of AD, demonstrating the dependability of the model in accordance with OECD standards (Figure 6).

### 3.5. Virtual Screening

**Figure 7:**
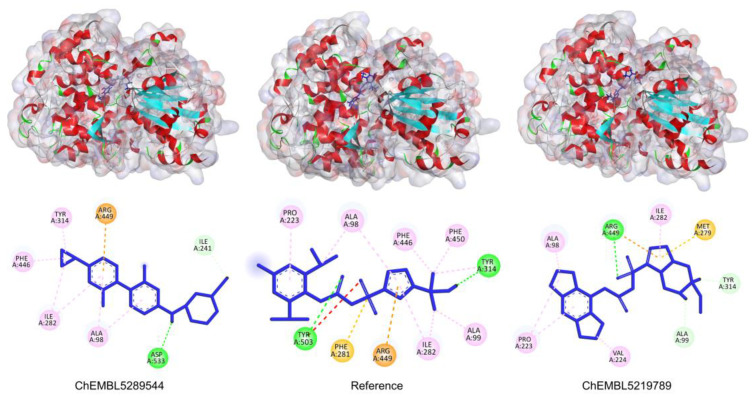
Two-dimensional illustration by Discovery studio visualizer. The reference and representative complexes (1 and 2) showed interactions with amino acid residues at the active site of NLRP3 where green: conventional hydrogen bond, light green: carbon–hydrogen bond, yellow: π-sulfur, orange: π-cation, pink: alkyl, light pink: π-alkyl bond.

The ligands with the lowest docking scores (S-scores), binding affinities, and promising binding poses were the best NLRP3 binders. All the compounds exhibited a unique interaction pattern, with hydrogen bonds involving Tyr314 and Tyr503 in the reference compound. Two compounds, ChEMBL IDs 5289544 and 5219789, with the best S-score of −7.5 and −8.2 kcal/mol, were selected. ChEMBL5289544 forms a hydrogen bond with Asp533, whereas ChEMBL5219789 forms a unique hydrogen bond with Met279 (Figure 7). Additionally, pi-cation interactions between the ligand’s aromatic rings and the positively charged Arg449 residue further stabilize the complexes. The presence of hydrophobic interactions with residues such as Phe446 and Ala98 further enhanced the binding affinity. Although all three complexes relied on hydrophobic interactions to stabilize the binding, the specific residues involved differed, as shown in Table 5.

### 3.6. MD Simulation Studies

**Figure 8:**
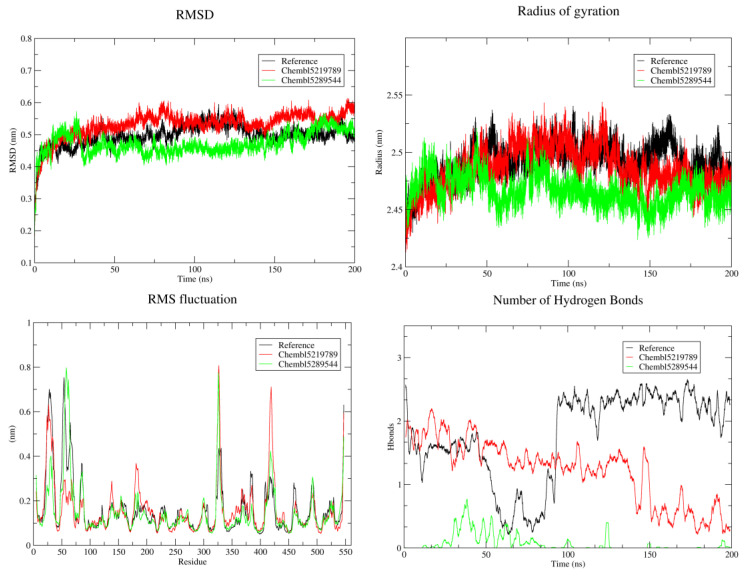
MD simulation of compounds during the 200 ns time period. The changes in RMSD, RMSF, Rg, and hydrogen bonding plots reveal the protein–ligand complex stability, flexibility, compactness, and interaction patterns.

MD was performed to investigate the conformational changes, stability, and flexibility of the protein–ligand system. The root mean square deviation (RMSD) of the backbone atoms was monitored over 200 ns of simulation time. Initially, all compounds exhibited a rapid increase in RMSD, indicating an equilibration phase of the system. As the system reached equilibrium, the RMSD plateau in ChEMBL5219789 increased from 0.4 to 0.56 Å, suggesting that the protein has attained a higher degree of flexibility and confirmation changes, whereas ChEMBL5289544 showed a decreased trend suggesting a gradual convergence towards a more stable conformation over time in comparison with the reference compound (Figure 8). The Rg values fluctuate over time, reflecting the dynamic nature of the protein. However, the overall trend indicated that the protein maintained a relatively stable conformation throughout the simulation. The average Rg value for the reference and ChEMBL5219789 compounds was approximately 2.47 nm, suggesting that the compact protein structure and binding of these ligands did not cause large conformational changes at the NLRP3 active site.

Regions with higher RMSF values indicated greater flexibility in the protein structure owing to the presence of disordered regions, as observed at various amino acid residues at 10-100, 330-340, and 400-450 in all complexes. These regions, identified by peaks in the RMSF plot, may correspond to functionally important regions of the protein, such as loops and hinges. The consistency of the RMSF profiles across the three simulations suggests that the observed flexibility is inherent to the protein structure and not an artifact of the simulation, although certain regions of the protein exhibit lower flexibility. A lower RMSF value indicates a rigid structure that helps maintain the overall stability of the protein. A higher number of hydrogen bonds were observed in both the reference and ChEMBL5219789, showing a greater propensity for hydrogen bonding interactions in comparison with ChEMBL5289544 (Figure 8).

### 3.7. MM/PBSA Studies

**Figure 9:**
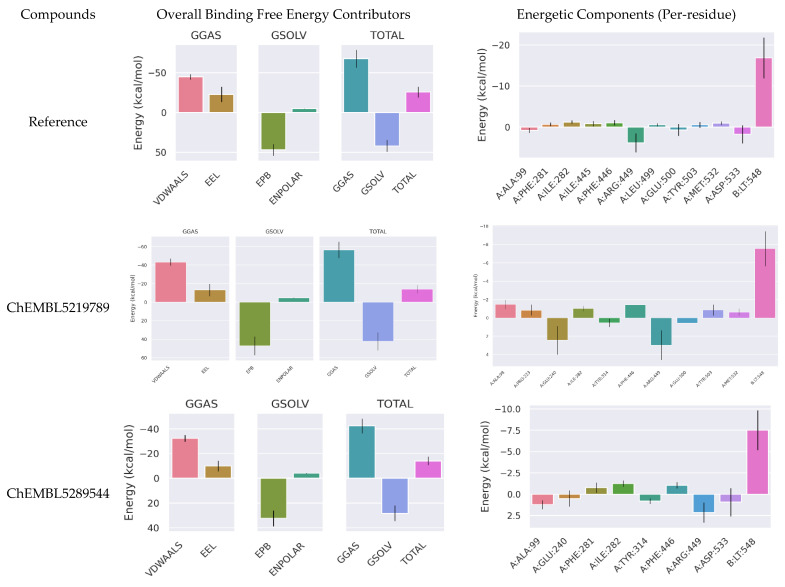
MM/PBSA analysis (**left**) binding free energy contribution by various interactions, (**right**) binding free energy contribution by active residues and ligand.

To gain deeper insight into the energetic contributions to ligand binding, MM/PBSA calculations were performed on a representative set of compounds (Table 6). The binding free energy is decomposed into the following constituents: van der Waals interactions (VDWAALSs), electrostatic interactions (EELs), polar solvation energy (EPB), and nonpolar solvation energy (ENPOLAR). Figure 9 illustrates the contributions of these energy terms to the overall binding free energy of the protein–ligand complexes, averaged over the last 200 frames of the MD simulation.

The calculated binding free energy (∆G_bind_) for the protein–ligand complex was found to be −25.68 kcal/mol (Reference), −14.06 kcal/mol (ChEMBL5219789), and −14.12 kcal/mol (ChEMBL5289544), indicating a favorable binding affinity. The decomposition of ∆G_bind_ revealed that van der Waals interactions (∆E_vdW_) and electrostatic interactions (∆E_elec_) were the primary driving forces for binding, contributing −43.4 and −13.06 kcal/mol and −32.31 and −10.04 kcal/mol for ChEMBL5219789 and ChEMBL5289544, respectively. The solvation energy (∆G_solv_) was also found to be significant, with a value of 42.2 and 28.23 kcal/mol.To identify the specific residues contributing to binding affinity, per-residue decomposition analysis was conducted. This analysis highlights the importance of residues such as Ala99, Ile282, Phe446, Tyr314, Arg449, Glu500, Tyr503, and Met532 in stabilizing the protein–ligand complexes. These residues form van der Waals interactions, electrostatic interactions, polar solvation energies, and nonpolar solvation energies with the ligand, contributing significantly to the overall binding energy.

## 4. Discussion

The release of cathepsins and MMPs by osteoclasts in response to NLRP3 activation may result in the degeneration of cartilage and bone [40]. Patients with RA may have elevated NLRP3 levels in the synovium; therefore, signaling compounds in the NLRP3 inflammasome pathway could be used as targets for RA treatment [10,41]. The ChEMBL database was utilized, and the initial dataset (529) was cleaned and filtered, resulting in a more reliable working dataset of 402 compounds to improve the quality and predictive power of the models. Compounds with ambiguous activity that could negatively impact classical and machine learning models were removed. By focusing on homogeneous datasets, the models were expected to learn robust patterns and make more accurate predictions. This dataset was used for the subsequent feature selection, model training, and evaluation. The observed differences between the active and inactive compounds have important implications for their biological activities. The high MW and lipophilicity of active compounds may influence their membrane permeability and interactions with biological targets. However, an increased number of rotatable bonds could also lead to conformational flexibility, potentially affecting binding affinity and selectivity. The higher TPSA of the active compounds suggests increased polarity, which may influence their solubility and bioavailability. A combination of physicochemical properties, rather than any single factor, likely contributed to the observed differences. Additionally, exploring the relationship between specific structural features and biological activity could provide valuable insights for future drug design and optimization.

PCA revealed that MW, LogP, and TPSA were the most influential parameters for determining the biological activity of the compounds. These properties are known to significantly influence drug absorption, distribution, metabolism, and excretion as well as the binding affinity to biological targets. MW can affect the bioavailability and permeability of compounds, whereas TPSA represents the polar surface area, and the number of hydrogen bond donors/acceptors can influence the interactions of compounds with biological targets and their solubilities. Identification of these key properties can aid in the rational design of new compounds with improved potency and selectivity. The subsequent principal components, although contributing less to the overall variance, provided valuable information regarding other relevant physicochemical properties. For example, the importance of hydrogen donors/acceptors in PC3 highlights their roles in molecular interactions and binding affinities. By understanding the complex interplay among these factors, researchers can optimize drug design strategies and develop effective therapeutic agents.

SAS maps are powerful cheminformatics tools based on systematic pairwise comparisons of all compounds based on their structural similarities and activities. SAS maps were visualized to understand the relationship between the structural similarities and activity differences [42]. The SAS map identified compounds exhibiting high similarity and low differences in activity, indicating that potential regions of the chemical space require optimization for enhanced activity and potency. These regions should be further explored to discover novel compounds with improved potency. Pairs with high similarity and activity differences represent activity cliffs that can provide insights into the molecular mechanisms underlying drug activity and guide the design of new, more potent compounds. A Murcko scaffold is a simplified representation of the core structure of a molecule. It is obtained by removing the side chains and heteroatoms, leaving behind the basic ring systems and their connections. The frequency of a Murcko scaffold refers to the number of compounds in a dataset that share the same core structure. The more frequently occurring scaffolds provided valuable insights into the structural requirements for inhibitory activity against NLRP3. The presence of tricyclic skeleton, aromatic rings, and heterocyclic systems in these scaffolds suggests that π-π interactions and hydrogen bonding may play a crucial role in protein–ligand binding. Additionally, the presence of functional groups, such as amines, amides, and carboxylic acids, indicates potential electrostatic interactions and hydrogen bond formation with key residues in the binding site. This knowledge can be used to design and synthesize novel compounds with improved potency and selectivity.

ML-enhanced QSAR models were developed to predict the inhibitory activity of NLRP3 inhibitors using molecular descriptors derived from MACCS and PubChem fingerprints. These ML techniques have transformed traditional QSAR analysis into a more powerful tool for predicting the effects of NLRP3 inhibitors, demonstrating how ML can improve classical chemometric methods. To enhance the traditional QSAR process, allowing for better prediction of inhibitory activity, feature selection, and more complex pattern recognition, machine learning models, kNN, GB, bootstrap aggregation, LightGBM, HGB, and RF were used. After molecular descriptor calculation, the Lazy Predict package was used to acquire robust ML models. Model performance was evaluated using cross-validation and test sets to ensure robust predictions. Among the top five best-performing models, the ensemble RF and bootstrap aggregating models showed relatively good mean absolute values compared to other models. RF trees have a high numerical value, which reduces overfitting and increases accuracy. Bootstrap aggregation focuses on lowering the variance rather than bias by increasing the randomness and combining multiple predictions. Multiple subsets of the original dataset were created by sampling with a replacement technique, with each subset used to train a decision tree model, and averaging all trees produced the final output [43]. Additionally, it can reduce overfitting and increase the accuracy and stability of the ML regression methods. RF is a modification of bootstrap aggregation that only considers a random subset of features compared to bootstrap aggregation, where each model takes all features [44].

Comparative analysis of the three protein–ligand complexes revealed distinct binding modes and key interactions. All three complexes exhibited a combination of hydrogen bonding, π-cation interactions, and hydrophobic interactions, which contributed to the overall binding affinity. However, the specific residues involved and the strength of these interactions vary across complexes. The stability of the protein structure was determined by RMSD analysis. The initial increase in RMSD during the equilibration phase was expected as the system relaxed to a stable conformation. The subsequent plateau phase indicated that the protein had reached a stable equilibrium state. The relatively moderate RMSD values observed throughout the simulation suggest that the protein maintains a well-defined structure that is essential for its biological function. The relatively stable Rg values observed in the simulations suggested that the protein maintained its overall folding and compactness throughout the simulation. Fluctuations in Rg can be attributed to the inherent flexibility of the protein, particularly in regions with high structural flexibility. However, the core structure of the protein remains relatively stable, as evidenced by the limited variation in Rg.

RMSF analysis provides valuable insights into the dynamic behavior of proteins. The identification of flexible regions can help understand the functional mechanisms of proteins and their interactions with other molecules. For example, flexible loops may play a role in ligand-binding or protein–protein interactions. The observed differences in flexibility between the different regions of the protein can also be attributed to their structural and functional roles. Regions involved in protein–protein interactions or ligand binding may exhibit lower flexibility to maintain specific interactions. In contrast, regions involved in conformational changes or dynamic processes may exhibit greater flexibility.

Analysis of hydrogen bond formation provides valuable insights into the stability and function of proteins. A stable hydrogen bond network is crucial for maintaining the structure of a protein and facilitating its interactions with other molecules. The fluctuations in the number of hydrogen bonds observed in the simulations reflect the dynamic nature of protein–ligand interactions. The identification of key hydrogen bonds consistently formed throughout the simulation can help understand the functional mechanism of the protein. These hydrogen bonds may play a critical role in stabilizing protein structure and facilitating ligand-binding interactions. MM/PBSA revealed the dominant contributions of VDWAALSs and EELs to the binding free energy, which was consistent with the observed hydrogen bonding and hydrophobic interactions between the protein and ligands. Targeting key residues identified in the per-residue decomposition analysis may enable the development of novel compounds with improved binding affinity and selectivity. These results provide valuable insights into the molecular mechanisms underlying protein–ligand interactions and can guide future drug design efforts. This study provides a deeper understanding of the molecular basis of ligand binding and enables the design of more potent and selective NLRP3 inhibitors.

## 5. Conclusions

This study employed a multidisciplinary in silico approach to investigate the structure–activity relationships of inhibitors targeting the NLRP3 inflammasome. Machine learning models were trained on a dataset with known bioactivity to predict potential inhibitory activity. Through the application of SALI, novel chemical scaffolds with potential inhibitory activities were identified. Molecular docking simulations provided insights into the binding modes of the ligands to the NLRP3 protein, highlighting the key interactions that contribute to their inhibitory activity. MD simulations further elucidated the stability and dynamics of the protein–ligand complexes, providing valuable information for the optimization of lead compounds. MM/PBSA calculations with per-residue decomposition analysis revealed valuable insights into the molecular mechanisms underlying protein–ligand interactions and the identification of potential targets for drug design. These results offer a promising avenue for the development of novel and potent NLRP3 inhibitors that can be used to treat inflammatory diseases, such as RA. Although classical methods (SAS maps, SALI, docking, MD, and MM/PBSA) offer detailed insights into molecular interactions and dynamics, they may have limited accuracy in predicting complex biological behaviors, require significant computational resources, and often lack the ability to model nonlinear or highly complex patterns that ML can capture. ML-based QSAR methods can improve prediction accuracy and handle large, complex datasets, but can suffer from overfitting and dependence on data quality and computational resources. These results demonstrated the potential of computational approaches for accelerating drug discovery. By combining these techniques, several promising compounds can serve as starting points for the development of novel NLRP3 inhibitors. Further in vitro validation is necessary to confirm the efficacy and safety of these compounds. The size of the dataset was not sufficiently large to comprehend additional areas of the chemical space. Moreover, the ChEMBL database generally lacks valuable scaffold information, which was the main limitation of this study. These findings contribute to the development of more robust predictive models and inform the design of more effective RA therapeutics.

## Figures and Tables

**Table 1:** Exploratory data analysis (EDA) of six drug-like descriptors and comparison between NLRP3 active (A1) and inactive (A2) compounds.

Descriptor	A1	A2	A1	A2	A1	A2	A1	A2	A1	A2	A1	A2
	MW		LogP		nHA		nHD		nRot		TPSA	
*p*-value	5.86 × 10^−13^		2.74 × 10^−1^		5.06 × 10^−7^		8.12 × 10^−7^		5.33 × 10^−1^		1.10 × 10^−15^	
Min	241.29	126.11	578.00	700.77	399.46	325.29	400.68	340.85	0.11	0.96	0.09	2.46
Max	−0.47	−1.30	6.27	7.33	3.18	2.95	3.13	3.01	−0.10	0.03	1.12	1.78
Median	1.00	1.00	10.00	11.00	5.00	4.00	5.07	4.18	0.29	0.78	0.09	1.00
Mean	0.00	0.00	4.00	4.00	2.00	2.00	1.96	1.53	−0.49	−0.11	1.44	−0.38
Skew	0.00	0.00	9.00	10.00	4.00	4.00	3.92	4.02	0.67	0.80	2.44	1.50
Kurtosis	29.60	12.47	155.76	195.18	88.41	67.43	90.24	69.75	0.01	1.10	−0.02	4.17

**Table 2:** PCA of the eigenvalues and cumulative variance (%) of the six physicochemical properties.

Property	PC1	PC2	PC3
MW	0.54	0.25	0.05
LogP	0.05	0.67	0.46
nHD	0.12	–0.51	0.80
nHA	0.53	–0.11	–0.37
nRot	0.33	0.35	0.14
TPSA	0.54	–0.31	0.01
Cumulative Variance (%)	0.44	0.69	0.84

**Table 3:** NLRP3 inhibitor frequency and chemotype analysis of top Murcko scaffolds.

ID	Scaffold	Frequency
ChEMBL5196575	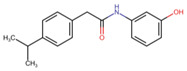	41
ChEMBL4795130	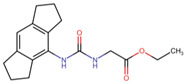	12
ChEMBL4204644	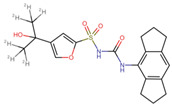	11
ChEMBL4759516	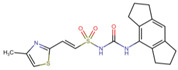	10
ChEMBL474722	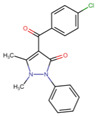	8
ChEMBL5172161	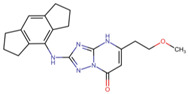	7

**Table 4:** Summary of evaluation metrics of regression algorithms built using PubChem and MACCS fingerprints.

Fingerprints	Models	RMSE (Train)	R^2^ (Train)	RMSE (Test)	R^2^ (Test)	Q^2^ (CV)	MAPE (Train)	MAPE (Test)
PubChem	Gradient Boosting	0.434	0.837	0.758	0.593	0.482	5.818	10.076
	k-Nearest Neighbors	0.516	0.768	0.732	0.620	0.383	7.115	9.167
	LightGBM	0.412	0.853	0.772	0.578	0.463	5.493	10.421
	Histogram Gradient Boosting	0.412	0.853	0.772	0.578	0.463	5.493	10.421
	Random Forest	0.365	0.884	0.731	0.622	0.465	4.610	8.988
MACCS	Gradient Boosting	0.483	0.797	0.714	0.639	0.484	6.542	9.663
	Bootstrap Aggregating	0.393	0.866	0.687	0.666	0.463	5.221	9.216
	LightGBM	0.414	0.851	0.703	0.650	0.477	5.340	9.556
	Histogram Gradient Boosting	0.414	0.851	0.703	0.650	0.477	5.340	9.556
	Random Forest	0.541	0.746	0.701	0.652	0.483	7.524	9.329

**Table 5:** Docked interaction analysis of NLRP3 protein with ligands.

Compounds	H Bonds	C-H Bond	Pi-Alkyl	Pi-Sulfur
Reference	Tyr314, Tyr503	-	Ala98, Ala99, Pro223, Ile282, Phe446, Phe450	Phe281, Arg449
ChEMBL5289544	Asp533	Ile241	Ala98, Ile282, Tyr314, Phe446	Arg449
ChEMBL5219789	Arg499	Ala99	Ala98, Pro223, Ile282, Val224	Arg449, Met279

**Table 6:** Binding free energy (kcal/mol) calculations using MM/PBSA.

Compounds	VDWAALSs	EELs	EPB	ENPOLAR	GGAS	GSOLV	TOTAL
Reference	−44.91 ± 3.4	−22.82 ± 9.66	47.05 ± 7.45	−5.01 ± 0.16	−67.72 ± 11.27	42.05 ± 7.4	−25.68 ± 6.82
5219789	−43.4 ± 3.77	−13.06 ± 6.62	47.08 ± 9.94	−4.67 ± 0.16	−56.46 ± 8.79	42.4 ± 9.83	−14.06 ± 4.53
5289544	−32.31 ± 2.72	−10.04 ± 4.26	32.41 ± 6.38	−4.19 ± 0.2	−42.35 ± 5.95	28.23 ± 6.28	−14.12 ± 3.51

Calculations represent average ± std. deviation.

## Data Availability

The dataset used in this study is available at “URL https://github.com/SI319 (accessed on 24 August 2024)”.

## Supplementary Materials

The following supporting information can be downloaded at https://www.mdpi.com/article/10.3390/cells14010027/s1: Table S1: Comparison of PubChem and MACCS fingerprints of compounds based on similarity, max values, and activity difference; Table S2: Lazy Predict was used to evaluate and fit all machine learning model using PubChem fingerprints; Table S3: Lazy Predict was used to evaluate and fit all machine learning model using MACCS fingerprints; Table S4: PubChem and MACCS hyperparameter tuning for the selected models; Figure S1: Representation of Murcko frequency and pIC_50_ values. The color of the dots represents pIC_50_, with a color change from red to blue indicating low to high pIC_50_ values. The size of the ball indicates Murcko frequency; Figure S2: The relationship between structure–activity landscape index (SALI) and pIC_50_ is shown, where the dot color represents pIC_50_, with blue to red color indicating low to high pIC_50_ values; Figure S3: The Y-scrambling test showed experimental and predictive measurement of the QSAR model.
