# Supplementary material for: Harnessing the Power of Machine Learning Guided Discovery of NLRP3 Inhibitors Towards the Effective Treatment of Rheumatoid Arthritis
Source: Cells. 2024 Dec 30;14(1):27. doi: 10.3390/cells14010027 (PMC11719798; doi:10.3390/cells14010027)
Supplement: Supplementary file 1 [file cells-14-00027-s001.zip › cells-3363055-supplementary.pdf]

Cells **2024**

<Supplementary Material>

Harnessing the Power of Machine Learning Guided Discovery of NLRP3 Inhibitors Towards the Effective Treatment of Rheumatoid Arthritis

Sidra Ilyas[1†], Abdul Manan[2†], Hee-Geun Jo [1,3*] and Donghun Lee [1*]

[1] Department of Herbal Pharmacology, College of Korean Medicine, Gachon University, 1342 Seongnamdae-ro, Sujeong-gu, Seongnam-si, 13120, Republic of Korea; sidrailyas6@gachon.ac.kr (S.I.)

[2] Department of Molecular Science and Technology, Ajou University, Suwon 16499, Korea; mananriaz012@gmail.com (A.M.)

[3] Naturalis Inc. 6, Daewangpangyo-ro, Bundang-gu, Seongnam-si 13549, Republic of Korea

* Correspondence: jho3366@hanmail.net (H.-G.J.); dlee@gachon.ac.kr (D.L.)

† These authors contributed equally to this work.

Supplementary Table S1. Comparison of PubChem and MACCS fingerprints of compounds based on similarity, max values, and activity difference.

**Fingerprints**	**Similarity**	**Activity Difference**	**Max Values**	**SALI**
**PubChem_ChEMBL ID**				
CHEMBL4637610_CHEMBL4647321	0.992	2.167491	8.167491	270.9364
CHEMBL5196575_CHEMBL5190030	0.9851852	2.752518	6.752518	185.795
CHEMBL5219210_CHEMBL5219789	1	2.413734	8.267606	241.3734
CHEMBL5219210_CHEMBL5220779	0.984375	2.032185	7.886057	130.0598
CHEMBL5180041_CHEMBL5196600	0.967033	3.113509	7.113509	94.44312
**MACCS_ChEMBL ID**				
CHEMBL3183703_CHEMBL4786282	0.9852941	3.39794	8.09691	231.06
CHEMBL4204644_CHEMBL4786282	0.9852941	3.60206	8.30103	244.9401
CHEMBL5180041_CHEMBL5196600	1	3.113509	7.113509	311.3509
CHEMBL5186980_CHEMBL5182226	1	2.621239	6.621239	262.1239
CHEMBL5219210_CHEMBL5219789	1	2.413734	8.267606	241.3734

Supplementary Table S2. Lazy Predict was used to evaluate and fit all machine learning model using PubChem fingerprints.

**Model**	**Adjusted R-Squared**	**R-Squared**	**RMSE**	**Time Taken**
**RandomForestRegressor**	0.43	0.64	0.71	0.29
**GradientBoostingRegressor**	0.38	0.61	0.74	0.18
**HistGradientBoostingRegressor**	0.31	0.57	0.78	0.13
**KNeighborsRegressor**	0.26	0.54	0.81	0.02
**LGBMRegressor**	0.26	0.54	0.81	0.09
**AdaBoostRegressor**	0.24	0.53	0.82	0.20
**NuSVR**	0.21	0.51	0.83	0.05
**BaggingRegressor**	0.21	0.51	0.83	0.08
**XGBRegressor**	0.21	0.51	0.84	0.13
**SVR**	0.20	0.50	0.84	0.06
**ExtraTreesRegressor**	0.12	0.45	0.88	0.24

**Model**	**Adjusted R-Squared**	**R-Squared**	**RMSE**	**Time Taken**
**MLPRegressor**	0.02	0.39	0.93	0.49
**ExtraTreeRegressor**	0.00	0.38	0.94	0.04
**PoissonRegressor**	-0.00	0.37	0.94	0.02
**BayesianRidge**	-0.02	0.36	0.95	0.11
**RidgeCV**	-0.05	0.35	0.96	0.03
**SGDRegressor**	-0.05	0.34	0.96	0.03
**ElasticNetCV**	-0.06	0.34	0.97	0.24
**LassoCV**	-0.06	0.34	0.97	0.16
**LassoLarsCV**	-0.06	0.34	0.97	0.07
**OrthogonalMatchingPursuitCV**	-0.07	0.33	0.97	0.02
**LassoLarsIC**	-0.08	0.33	0.98	0.03
**Ridge**	-0.08	0.32	0.98	0.01
**LinearRegression**	-0.09	0.32	0.98	0.07
**TransformedTargetRegressor**	-0.09	0.32	0.98	0.02
**DecisionTreeRegressor**	-0.11	0.30	0.99	0.05
**TweedieRegressor**	-0.11	0.30	0.99	0.02
**HuberRegressor**	-0.13	0.29	1.00	0.04
**GammaRegressor**	-0.14	0.29	1.00	0.02
**LarsCV**	-0.20	0.25	1.03	0.08
**LinearSVR**	-0.20	0.25	1.03	0.05
**OrthogonalMatchingPursuit**	-0.26	0.21	1.05	0.02
**Lars**	-0.45	0.09	1.13	0.05
**PassiveAggressiveRegressor**	-0.48	0.08	1.14	0.02

**Model**	**Adjusted R-Squared**	**R-Squared**	**RMSE**	**Time Taken**
**ElasticNet**	-0.61	-0.00	1.19	0.02
**LassoLars**	-0.64	-0.03	1.20	0.01
**DummyRegressor**	-0.64	-0.03	1.20	0.05
**Lasso**	-0.64	-0.03	1.20	0.02
**RANSACRegressor**	-0.67	-0.05	1.22	0.19
**QuantileRegressor**	-0.68	-0.05	1.22	0.04
**GaussianProcessRegressor**	-19.46	-11.79	4.25	0.13
**KernelRidge**	-34.61	-21.25	5.61	0.14

Supplementary Table S3. Lazy Predict was used to evaluate and fit all machine learning model using MACCS fingerprints.

**Model**	**Adjusted R-Squared**	**R-Squared**	**RMSE**	**Time Taken**
**RandomForestRegressor**	0.46	0.66	0.69	0.28
**BaggingRegressor**	0.43	0.64	0.71	0.05
**GradientBoostingRegressor**	0.42	0.64	0.72	0.19
**HistGradientBoostingRegressor**	0.41	0.63	0.72	0.14
**LGBMRegressor**	0.39	0.62	0.73	0.09
**KNeighborsRegressor**	0.39	0.62	0.73	0.02
**XGBRegressor**	0.34	0.58	0.77	0.18
**AdaBoostRegressor**	0.33	0.58	0.77	0.12
**ExtraTreesRegressor**	0.30	0.56	0.79	0.22
**ExtraTreeRegressor**	0.30	0.56	0.79	0.02
**NuSVR**	0.28	0.55	0.80	0.04
**MLPRegressor**	0.27	0.54	0.80	0.51

**Model**	**Adjusted R-Squared**	**R-Squared**	**RMSE**	**Time Taken**
**SVR**	0.26	0.54	0.81	0.03
**PoissonRegressor**	0.14	0.46	0.87	0.02
**SGDRegressor**	0.11	0.45	0.88	0.02
**RidgeCV**	0.11	0.44	0.89	0.03
**Ridge**	0.11	0.44	0.89	0.01
**LinearRegression**	0.11	0.44	0.89	0.09
**TransformedTargetRegressor**	0.11	0.44	0.89	0.02
**LassoLarsIC**	0.10	0.44	0.89	0.03
**BayesianRidge**	0.10	0.44	0.89	0.02
**LassoCV**	0.10	0.44	0.89	0.14
**LassoLarsCV**	0.10	0.44	0.89	0.06
**ElasticNetCV**	0.10	0.44	0.89	0.15
**DecisionTreeRegressor**	0.08	0.43	0.90	0.04
**GammaRegressor**	0.01	0.38	0.94	0.02
**LinearSVR**	-0.00	0.37	0.94	0.04
**TweedieRegressor**	-0.00	0.37	0.94	0.02
**HuberRegressor**	-0.03	0.36	0.95	0.04
**OrthogonalMatchingPursuit**	-0.16	0.28	1.01	0.02
**PassiveAggressiveRegressor**	-0.19	0.26	1.02	0.02
**LarsCV**	-0.19	0.25	1.03	0.07
**OrthogonalMatchingPursuitCV**	-0.22	0.24	1.04	0.02
**ElasticNet**	-0.60	-0.00	1.19	0.02
**DummyRegressor**	-0.64	-0.03	1.20	0.02

**Model**	**Adjusted R-Squared**	**R-Squared**	**RMSE**	**Time Taken**
**LassoLars**	-0.64	-0.03	1.20	0.02
**Lasso**	-0.64	-0.03	1.20	0.02
**QuantileRegressor**	-0.68	-0.05	1.22	0.04
**RANSACRegressor**	-2.11	-0.94	1.66	0.20
**Lars**	-2.28	-1.05	1.70	0.04
**GaussianProcessRegressor**	-18.51	-11.19	4.15	0.11
**KernelRidge**	-34.49	-21.18	5.60	0.13

Supplementary Table S4. PubChem and MACCS hyperparameter tuning for the selected models.

**Fingerprinting**	**Models**	**Hyperparameters**
PubChem	Gradient Boosting	{'learning_rate '0.1, 'n_estimators' 200}
	k-Nearest Neighbors	{'n_neighbors' 3}
	Light GBM	{'learning_rate' 0.05, 'n_estimators' 500}
	HistGradientBoosting	{'learning_rate' 0.05, 'n_estimators' 500}
	Random Forest	{'max_depth' None, 'n_estimators' 100}
MACCS	Gradient Boosting	{'learning_rate'0.1, 'n_estimators' 100}
	Bagging	{'max_samples'0.75, 'n_estimators'500}
	LightGBM	{'learning_rate' 0.1, 'n_estimators'200}
	HistGradientBoosting	{'learning_rate' 0.1, 'max_iter'200}
	Random Forest	{'max_depth' 5, 'n_estimators'200}

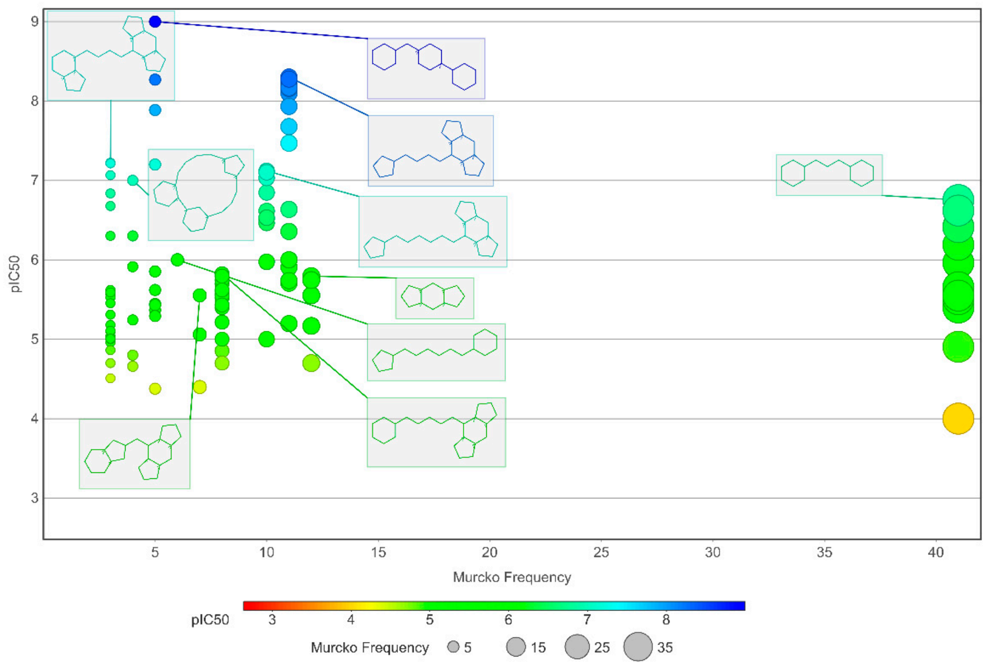

Supplementary Figure S1. Representation of Murcko frequency and pIC_{50} values. The color of the dots represents pIC_{50}, with a color change from red to blue indicating low to high pIC_{50} values. The size of the ball indicates Murcko frequency.

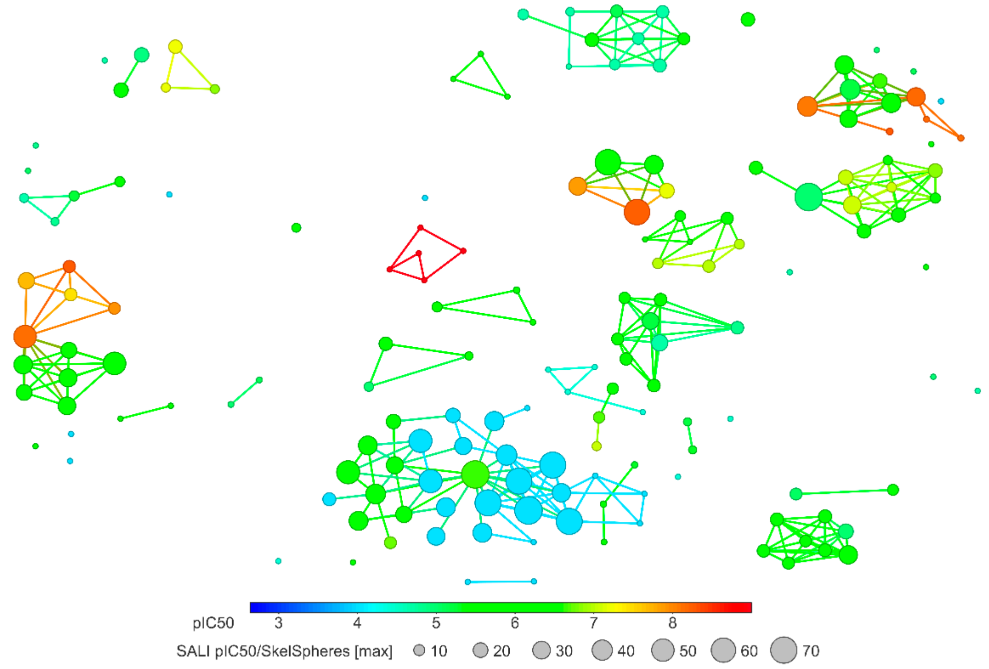

Supplementary Figure S2. The relationship between structure–activity landscape index (SALI) and pIC_{50} is shown, where the dot color represents pIC_{50}, with blue to red color indicating low to high pIC_{50} values.

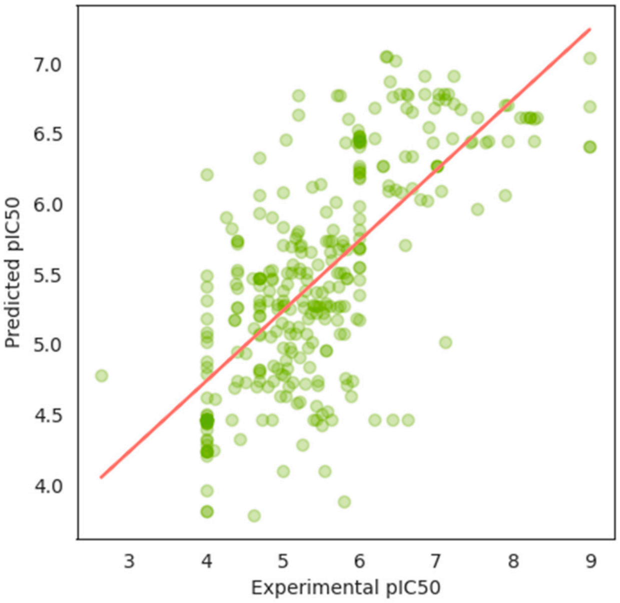

Supplementary Figure S3. The Y-scrambling test showed experimental and predictive measurement of the QSAR model.
